# Planned neck dissection following chemo-radiotherapy in advanced HNSCC

**DOI:** 10.1186/1477-7800-1-6

**Published:** 2004-09-17

**Authors:** Tejpal Gupta, Jai Prakash Agarwal

**Affiliations:** 1 Department of Radiation Oncology, Clinical Research Centre, Advanced Centre for Treatment Research & Education in Cancer (ACTREC), Tata Memorial Centre, Kharghar, Navi Mumbai: 410208, INDIA; 2 Department of Radiation Oncology, Tata Memorial Hospital, Parel, Mumbai: 400 012, INDIA

**Keywords:** chemo-radiotherapy, HNSCC, and neck dissection

## Abstract

**Background:**

Neck dissection has traditionally played an important role in the management of patients with regionally advanced head and neck squamous cell carcinoma (HNSCC) treated with radical radiotherapy alone. However, with the incorporation of chemotherapy in the therapeutic strategy for advanced HNSCC and resultant improvement in outcome the routine use of post chemo-radiotherapy neck dissection is being questioned.

**Methods:**

Published data for this review was identified by systematically searching MEDLINE, CANCERLIT & EMBASE databases from 1995 until date with restriction to the English language.

**Results:**

There is lack of high quality evidence on the role of planned neck dissection in advanced HNSCC treated with chemo-radiotherapy. A systematic literature search could identify only one small randomized controlled trial (Level I evidence) addressing this issue, albeit with major limitations. Upfront neck dissection followed by chemo-radiotherapy resulted in better disease-specific survival as compared to chemoradiation only. Several single arm prospective and retrospective reports were also identified with significant heterogeneity and often-contradictory conclusions.

**Conclusions:**

Planned neck dissection after radical chemo-radiotherapy achieves a high level of regional control, but its ultimate benefit is limited to a small subset of patients only. Unless there are better non-invasive ways to identify residual viable disease, the role of such neck dissection shall remain debatable. A large randomized controlled trial addressing this issue is needed to clarify its role and provide evidence-based answers.

## Introduction

**Figure 1:**
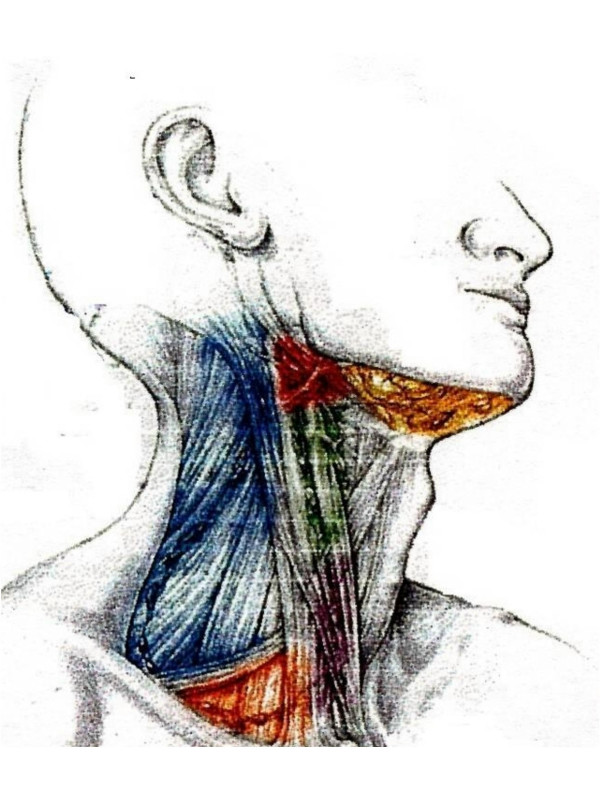
Sketch anatomy of standard neck incision

The optimal management of the neck in loco-regionally advanced head & neck squamous cell carcinomas (HNSCC) following primary chemo-radiotherapy remains controversial [1,2]. Traditionally neck dissection (Fig 1) was thought to improve neck control in patients with regionally advanced disease (N2–N3 disease) treated with radical radiotherapy alone [3,4]. However, with the incorporation of chemotherapy in the therapeutic strategy for advanced HNSCC and resultant improvement in outcome [5,6], the routine use of post chemo-radiotherapy neck dissection is being questioned [7,8]. Some authors recommend neck dissection for bulky nodal disease after chemo-radiation as part of organ preservation protocol in an elective manner, regardless of the response in the neck provided the primary is controlled. Others argue that it is an ineffective procedure and should be abandoned. Nevertheless, most investigators agree that elective neck dissection be performed for patients with less than a complete response in the neck after combined modality therapy to optimize regional control. This review attempts to provide the discerning reader a bird's eye view of the available evidence on this controversial issue.

## Methods

### Literature search strategy

Published data for this review was identified by systematically searching the MEDLINE, CANCERLIT & EMBASE databases from 1995 until date with restriction to the English language. "Head & Neck cancer" OR "HNSCC" was combined with "chemo-radiotherapy" OR "chemo-radiation" as Medical Subject Heading (MeSH) terms and each of the following phrase used as text words: "adjuvant neck dissection"; "planned neck dissection"; and "neck management". Relevant cross-references were also considered.

## Results & Discussion

### The evidence

There is only one small randomized control trial (American Society of Clinical Oncology [9] Level I evidence) evaluating the role of planned neck dissection in advanced HNSCC treated with primary chemo-radiotherapy. Carinci [10] et al randomly assigned patients with advanced unresectable HNSCC to either elective neck dissection followed by chemo-radiotherapy (Group I, n = 23) or chemo-radiotherapy alone (Group II, n = 31). The two groups were reasonably well balanced for known prognostic factors. The 2-and 5-year disease-specific survival rates significantly favored the surgical arm (52% and 26% for Group I versus 29% and 0% for Group II respectively). A Cox regression analysis adjusted for T-stage, N-stage, age and gender showed that only therapy (Group I versus II) reached a positive and significant odds ratio in association with the probability of death (p = 0.0366 in favor of neck dissection). This study however suffers from major limitations. Firstly, the trial methodology was not detailed adequately to assess the validity of the interpretations. The investigators neither specified the method of randomization (why was the distribution unequal in the two arms) nor about stratification on known prognostic factors. Secondly, the numbers of patients in each arm were too small to draw any definite conclusions without ruling out an element of bias. Thirdly, the radiotherapy delivery was suboptimal (only 60–65 Gy with conventional fractionation) for sterilizing advanced HNSCC, in which case the addition of neck dissection was expected to improve outcome. Finally, since neck dissection was done upfront rather than after chemo-radiotherapy, the results of this trial cannot be directly extrapolated to the issue under consideration.

In absence of high quality evidence, the best available evidence tempered with clinical judgment often guides decision-making. Two of the recently published reports [11,12] somewhat at contradiction with each other are briefly discussed to illustrate the dilemma.

Argiris [11] et al evaluated 131 patients with HNSCC having N2–N3 disease treated on concurrent chemo-radiotherapy protocols. Neck dissection was performed in 92 (70%) patients, either before (n = 31) or after chemo-radiotherapy (n = 61). With a median follow-up of 4.6 years, the 5-year loco-regional progression-free-survival (PFS) was significantly better in patients with planned neck dissection as compared to those without neck dissection (88% versus 74% respectively, p = 0.02) The addition of neck dissection to chemo-radiotherapy resulted in only one neck failure in 92 patients (neck PFS 99%) versus six neck failures in 39 patients (neck PFS 82%) not undergoing neck dissection (p = 0.0007). Neck dissection was however, not beneficial in patients with a complete clinical response (CCR). Of the 92 patients with a CCR, 62 underwent neck dissection, of which only 1 relapsed in the neck (neck PFS 98%). The neck PFS of 92% (2 neck failures) in the 30 patients in CCR who did not undergo neck dissection was not significantly different (p = 0.21). On subset analysis, in patients with N3 disease (n = 27), there was either a trend or a statistically significant advantage in all the survival parameters for the neck dissection arm. In contrast, in patients with N2 disease (n = 104), only the neck control improved with neck dissection. The local PFS, distant PFS, and the overall survival were similar irrespective of neck dissection. The authors concluded that in patients with N3 stage and less than CCR it was necessary to add neck dissection for optimal disease control, whereas in patients with N2 disease in CCR, neck dissection could safely be omitted without compromising outcome.

Brizel [12] et al identified 108 patients with nodal disease from a cohort of 154 patients on concurrent chemo-radiation protocols. A modified neck dissection was performed in 65 (60%) of 108 patients. With a median follow up of 4 years for surviving patients, the neck control rate was 100% for N1 patients irrespective of neck dissection being performed or not. Their disease-free-survival (DFS) was 70% with no differences relative to neck dissection. In N2–N3 patients, a CCR was achieved in 43 (55%) patients. Ten patients with local progression or systemic dissemination were excluded from analysis. Of the 52 patients undergoing neck dissection in N2–N3 group, only 1 regional relapse was seen, in contrast to 3 neck failures out of 16 in those not undergoing dissection (p = 0.05). The 4-year DFS was 75% for N2–N3 patients with a CCR and neck dissection versus 53% for those with CCR but no neck dissection (p = 0.08). The 4-year overall survival was also better for the dissection arm (77% versus 50% respectively, p = 0.04). The authors concluded that the policy of neck dissection in patients with N2–N3 disease even in CCR is justified to optimize loco-regional control and survival.

Apart from the afore-mentioned two reports, there are a few reasonably large studies (involving >50 patients: Table 1) and several smaller ones, both prospective and retrospective published in the last decade trying to define the benefit of such intervention with conflicting results [2,7,13-24]. However, significant heterogeneity in selection criteria as well as variable treatment schedules and response assessment methodology amongst these reports introduces a great deal of bias precluding any definitive conclusions.

**Table 1:** Neck failure in selected series of chemo-radiotherapy for HNSCC treated with or without ND

Author (year)	No of pts (n)	Pts in CCR	Neck failures (overall)		Neck failure (pts in CCR)		Remark(s)
			ND done	ND not done	ND done	ND not done	
McHam^2 ^(2003)	N2–N3: 109	65	5/76	4/33	1/32	4/33	ND needed for all N2–N3 patients
Grabenbauer^7 ^(2003)	N0–N3: 142	97	Only patients with CCR offered ND		9/56	4/41	No clear benefit of ND after CCR
Clayman^13 ^(2001)	N2–N3: 66	29	5/18	6/48	0/4	0/25	ND not needed for patients in CCR
Stenson^14 ^(2000)	N2–N3: 69	30	1/69	NA	0/30	NA	All 69 pts had ND; needed for N2–N3
Robbins^15 ^(1999)	N2–N3: 52 (56 heminecks)	33	1/34	2/20	0/16	0/17	Good control with ND for N2–N3
Lavertu^16 ^(1999)	N1–N3: 78	55	8/78 neck failures in all		NK	NK	ND done for all pts with N2–N3
Lavertu^17 ^(1997)	N0–N1: 47	43	0/6	4/38	0/5	4/38	ND needed for all N2–N3 patients even in CCR
	N2–N3: 53	30	1/35	3/12	0/18	3/12	

ND = neck dissection; CCR = clinical complete response; pts = patients; NA = not applicable; NK = not known

The potential benefit of planned neck dissection after a course of intensive chemo-radiotherapy in terms of improved regional control with or without an impact on survival needs to be weighed against the expected morbidity associated with the surgical procedure [12,14,25]. One argument put forward in favor of planned neck dissection even for patients in CCR is the high rate of pathological positivity (30%–50%) depending upon the meticulousness of sectioning by the pathologist [14,17]. However, a significant majority of them actually may represent microscopic non-viable residual disease only, as has been demonstrated by Strasser using Ki-67 proliferating index [26], unlikely to relapse later. Proponents of neck dissection also argue that the ultimate success rate of salvage neck dissection after a relapse in the neck treated with full dose chemo-radiotherapy is small, whereas the morbidity is high [10,25]. In contrast, the morbidity of a planned neck dissection is at best modest, when scheduled between 6–12 weeks from end of chemo-radiotherapy [7,14,17], which is supposed to be the time window between acute and chronic radiation injury.

## Conclusions & Recommendations

Planned neck dissection after radical chemo-radiotherapy achieves a high level of regional control, but its ultimate benefit is limited to a small subset of patients only. The morbidity of such dissection is small, but significant. Its impact on survival is yet to be completely realized. In the majority of patients it is either unnecessary because there is no residual disease in the neck or futile because of unsalvageable primary recurrence or distant metastases. Nevertheless, it is recommended that planned neck dissection be performed for patients with less than a complete response in the neck after combined modality therapy to optimize regional control, provided the primary is controlled and there is no evidence of distant metastases. It should also be performed as part of salvage surgery for locally persistent or residual disease at primary site. The criterion for planned neck dissection for patients with advanced nodal disease with a CCR in the neck following chemo-radiotherapy should incorporate not only the nodal staging but also the actual size of the involved lymph nodes. Unless there are better non-invasive ways to identify residual viable disease, which could include functional imaging like Positron Emission Tomography and biological assays like hypoxia markers, the role of such neck dissection shall remain debatable. A large randomized controlled trial across several institutions addressing these issues is needed to clarify the role of planned neck dissection in advanced HNSCC treated with primary chemo-radiotherapy and provide evidence-based answers.

## Source of funding

No source of funding involved in this review

## Competing interest or Conflict of interest

None declared.

## Authors' Contributions

Dr JP proposed the idea of systematic review on the issue

Dr TG did the literature search & prepared the manuscript

Dr JP critically reviewed and revised the manuscript
